# Where there’s smoke, there’s fire: what current and future providers do and do not know about electronic cigarettes

**DOI:** 10.1186/s12889-020-09265-5

**Published:** 2020-07-20

**Authors:** Josephine Hwang, Crystal Lee, Eric Mastrolonardo, Rosemary Frasso

**Affiliations:** 1 Jefferson College of Population Health, Thomas Jefferson University, Philadelphia, PA USA; 2 Sidney Kimmel Medical College, Thomas Jefferson University, Philadelphia, PA USA

**Keywords:** Electronic cigarettes, Evidence-based knowledge, Healthcare professionals, Health consequences, Freelisting, Patient counseling, Curricular development

## Abstract

**Background:**

Health care providers play a pivotal role as educators on health-related matters ranging from vaccination to smoking cessation. With the rising popularity of electronic cigarettes (e-cigarettes), providers face a new challenge. To date, studies have identified a general lack of knowledge among providers regarding e-cigarettes and discomfort with counseling patients on e-cigarette use. This study aims to systematically explore the perspectives of different health care providers on e-cigarettes and their health implications. With a growing availability of research on the health consequences of e-cigarette use, our study also aims to assess the familiarity of our participants with this literature.

**Methods:**

From July to October 2018, a sample of attendings (*n* = 15), residents (n = 15), medical students (*n* = 33), and nursing students (*n* = 28) from Thomas Jefferson University participated in a freelisting interview and survey.

**Results:**

Our study found that perceptions of e-cigarettes vary across different participant groups, as evidenced by the range of responses when asked to think about e-cigarettes and their health implications. We identified gaps in knowledge among students regarding FDA regulation of e-cigarettes and found that attending physicians are less aware than junior trainees of the prevalence of use. Familiarity with evidence-based health consequences was variable and low across all groups. Finally, participants most commonly reported learning about e-cigarettes from news outlets and social media rather than professional platforms.

**Conclusion:**

This study highlights the need for curricular development in nursing and medical schools, residency training, and continuing medical education regarding e-cigarette use and their impact on human health.

## Background

Electronic cigarettes, otherwise known as e-cigarettes, vapes or Electronic Nicotine Delivery Systems (ENDS), are tobacco products introduced to the United States in 2006, initially marketed as cigarette alternatives [1]. These battery-powered devices, with over 460 brands, contain liquids that are aerosolized by a coiled heating component [2]. Today, these devices are utilized not only by current cigarette users but increasingly by smoke-naïve individuals as well. The prevalence of e-cigarette use among middle and high school students has increased nearly ten-fold over 4 years [3]. In fact, e-cigarettes have been the leading tobacco product used by this age group since 2014, with 1 in 4 students reporting use [4].

While data confirm that e-cigarette use is less harmful than combustible cigarettes (c-cigarettes), the product is by no means safe. Nearly all e-cigarettes contain nicotine, with concentrations ranging from 45 to 131% [5]. Blood nicotine levels in e-cigarette users are variable and can be comparable to those in cigarette smokers [6, 7]. This is particularly concerning given the prevalence of e-cigarette use among youth as nicotine has been shown to interfere with adolescent brain maturation, altering risk-taking and reward-seeking behaviors and increasing risk of long-term impairment in attention and cognition [3, 8, 9].

American youth overall are more likely than adults to use e-cigarettes, and studies have shown that use in this age group is associated with increased odds of experimentation with c-cigarettes as well as increased future c-cigarette use [6, 10–13].

Although longitudinal data are still being collected, vaporized particles (aerosols) emitted from e-cigarettes contain some of the same carcinogens found in c-cigarettes, including acetaldehyde, formaldehyde, and particulates, as well as heavy metals like lead and chromium [14–16]. E-cigarette aerosols have been linked to DNA damage and pro-inflammatory effects in in vivo studies [15, 17–20].

Evidence has shown that individuals who substitute e-cigarette for c-cigarettes have less exposure to harmful toxic substances [21, 22]. However, the efficacy of e-cigarettes as a smoking cessation tool remains inconclusive and the U.S. Food and Drug Administration (FDA) has yet to approve them for this use [12, 23–28].

As e-cigarette use increases, providers (physicians and nurses) need to be prepared to provide council and education. Among providers, self-reported general knowledge is low and often limited to e-cigarettes as a cessation aid [29–35]. To our knowledge, little is known about providers’ understanding of the health implication of use outside of the context of cessation.

Our overarching goal was to identify specific gaps in knowledge that could hinder efforts to council patients effectively in both practicing and future providers. Study findings may be used to inform educational interventions that would prepare providers to address the growing use of these harmful products among a new generation. We predicted that current and future providers’ knowledge would mirror that of the lay community, in that it is limited, varied, and not nested in evidence.

## Methods

### Study setting

This study was conducted at Thomas Jefferson University and Thomas Jefferson University Hospital (TJU and TJUH, respectfully) in Philadelphia, Pennsylvania.

### Study design and sample

We engaged the Jefferson healthcare community using the freelisting interview approach and surveys to examine shared and divergent perceptions of e-cigarettes and their health implications among attending physicians, residents, medical students, and nursing students. Freelisting is a standard qualitative research approach used to define elements of a domain (e.g. health implications of e-cigarette use) and measure the extent to which members of a group (e.g. attending physicians) share those perceptions [36]. Free listing has been used to explore topics ranging from shared decision-making, early psychosis to pediatric head injury [37–40].

We employed a purposeful sampling approach to engage primary care providers, nursing students, and medical students employed or studying at TJUH and TJU between June 2018 and October 2018. All participants were given the opportunity to decline participation or terminate their participation in the study prior to the dissemination of results in November 2018. A total of 33 medical students, 28 nursing students, 15 resident physicians, and 15 attending physicians were interviewed following informed verbal consent. Each participant was offered a protein bar in compensation for their time. All study procedures were reviewed and approved by the TJU Institutional Review Board (IRB).

### Data collection

There were two steps to data collection, 1) freelisting interview and 2) a brief survey. Data were collected by the first author (JH) or two trained research assistants. Resident physicians, medical students, and nursing students were approached on Jefferson’s campus. Attending physicians were identified on the Thomas Jefferson University Hospital website and invited by email to schedule a session through an online scheduler. For the freelisting step, participants were asked to list words or phrases that came to mind when they thought about 1) e-cigarettes and 2) health implications of e-cigarette use. No limits were placed on the number of responses or the time needed to generate a list. The interviewer documented freelisting responses in order on paper data collection forms [See Additional File 1]. Survey questions, which are provided in an additional file, explored sources of e-cigarette-related information and evaluated familiarity with the evidence-based health implications of e-cigarette use, and demographic data were collected [See Additional File 1] [22]. Data collection took between 5 and 10 min.

### Analysis

Freelisting responses were reviewed by two members of the research team to standardize word forms by combining plural, singular, and synonymous words. For instance, the terms “teenagers,” “teens,” “youth,” and “kids” were categorized as “young people.” Responses that were given as longer phrases were truncated when categorized additionally, brand names were replaced with [Withheld] in the text and figures. An original version of each participant’s list was kept and revisited to ensure the intended meaning was preserved after data cleaning. Standardized terms for each group were entered into Anthropac Version 1.0 (Analytic Technologies, 2003), a software that calculates salience index (Smith’s *S*) of free lists. Salience characterizes terms that are prototypes for the particular domain of interest that take into account the order of the term within each participant’s list as well as the frequency that the term appears across participants in the group. The calculated salience index is defined as *S*_*j*_ = [*∑* ((*L*_*i*_ − *R*_*j*_ + 1)/ *L*_*i*_)]/*N*, where *L*_*i*_ is the length of each list *i*, *R*_*j*_ is the rank of item *j* in list *i*, and *N* is the number of lists in the group [36]. The freelist terms for each group was then sorted highest to lowest salience. Following the example of prior studies that used the freelisting approach, a scree plot was generated with the x-axis representing each freelist term and the y-axis representing salience score [37, 38, 41, 42]. The plots were individually inspected for a natural inflection point, where the flattening of the slope indicated decreased frequency of terms within the respective group. Terms above the inflection point were retained as salient and sorted into Venn Diagrams to present similarities and divergences in salient concepts among the four groups (medical students, nursing students, residents, attendings).

Forced-choice survey responses were re-coded into either “Correct” or “Incorrect.” Likert-type responses “Agree” and “Strongly agree” were collapsed, as were “Disagree” and “Strongly disagree.” These responses were then re-coded into either “Correct” or “Incorrect” based on the 2018 consensus study report by the National Academies of Sciences, Engineering, and Medicine. The responses “I don’t know” were left as is. Analysis of survey responses was performed using SPSS Statistics© Version 24. Demographic data and sources of e-cigarette-related information were analyzed using basic descriptive statistics. Knowledge evaluation responses were analyzed using crosstabs to compare the frequency of correct and incorrect answers among attendings, residents, medical students, and nursing students. Raw data available upon request to corresponding author.

## Results

### Study population

A total of 91 participants (33 medical students, 28 nursing students, 15 resident physicians, 15 attending physicians) completed the free listing interview and survey questionnaire. Demographic characteristics are presented in Table 1.

**Table 1:** Age distribution of participants in each occupational group

Age Group	Attendings (n = 15)	Residents (n = 15)	Medical Students (n = 33)	Nursing Students (n = 28)
18–25	0	0	30	17
26–30	2	13	3	8
31–35	2	2	0	2
36–40	3	0	0	0
41–45	3	0	0	0
46+	5	0	0	1

### Comparing perspectives on E-cigarettes and related health implications

**Fig. 1:**
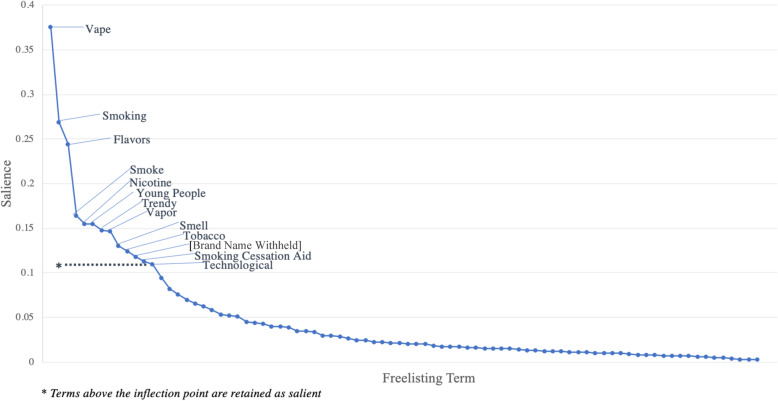
Freelisting responses by medical students when asked about electronic cigarettes. Each point along the x-axis represents a freelisting term, with the y-axis corresponding to the salience index, S

Salient terms for each question are organized by respondent occupation and summarized in an additional file [see Additional File 2]. Salience takes into account both frequency and rank, as well as the total number of free lists in that group. An example of a scree plot used to identify the most salient terms among a group of participants is shown in Fig. 1.

**Fig. 2:**
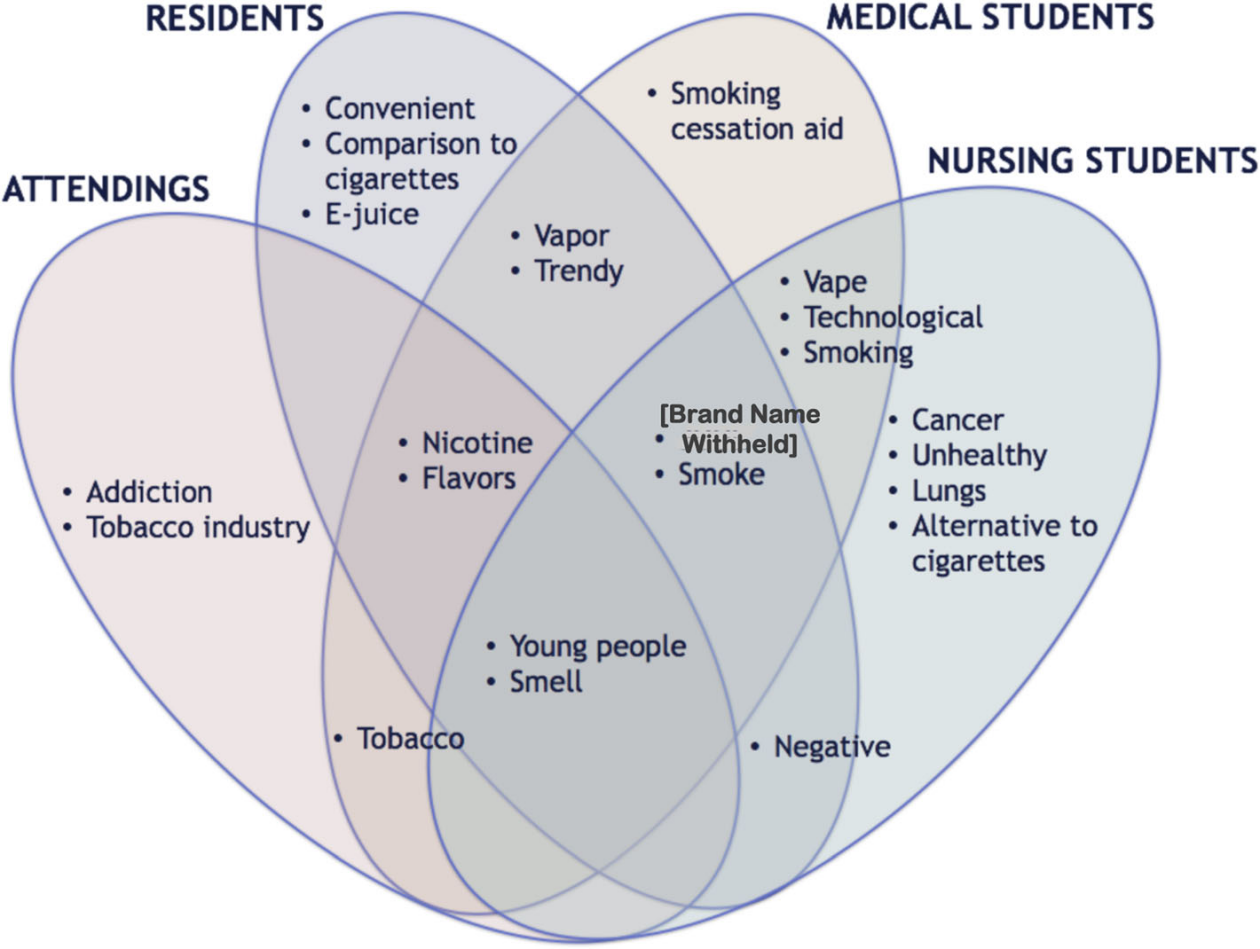
Comparison of attending, resident, medical student, and nursing student perspectives on electronic cigarettes

Salient responses to the prompt, “List all the words that come to mind when you of e-cigarettes” is summarized in Fig. 2. When asked what words or phrases come to mind when thinking about electronic cigarettes, “young people” and “smell” of e-cigarette aerosols were salient across all four groups. Terms shared between participants in the medical profession included “nicotine” and “flavors.” Terms shared between residents, medical students, and nursing students included a brand name [withheld]. “Trendy” and “vapor” were salient terms shared by residents and medical students. Both medical students and nursing students associated the devices with the words like “vape” and their “technological” design. Medical students also associated e-cigarettes with “smoking cessation aid,” while nursing students shared terms related to health, such as “cancer,” “lungs” and “unhealthy.” Other salient concepts for residents included the convenience of the devices, such as their portability and reusability, as well as comparisons to traditional cigarettes, in particular commenting that e-cigarettes are safer than cigarettes. Uniquely salient terms for attendings included “addiction” and “tobacco industry.”

**Fig. 3:**
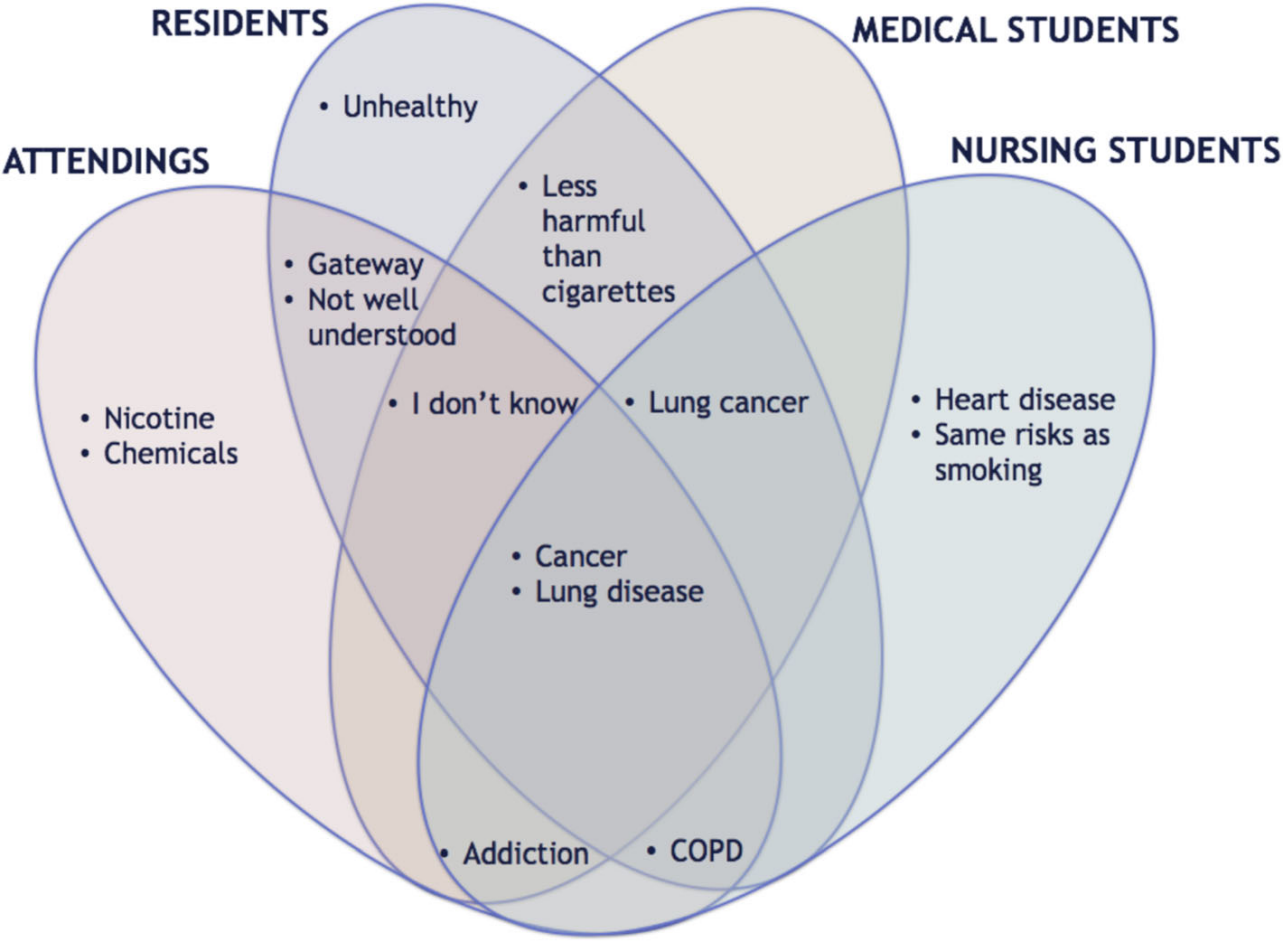
Comparison of attending, resident, medical student, and nursing student perspectives on the health implications of electronic cigarette use

Salient responses to the prompt, “List all the health implications that come to mind when you about e-cigarette use” is summarized in Fig. 3. When asked what words or phrases come to mind when thinking about the health implications of e-cigarette use, “cancer” and “lung disease” were the salient terms shared by all four groups. Both residents and attending physicians associated e-cigarettes with being a potential “gateway” for cigarette use and expressed that health implications of e-cigarette use are “not well understood.” The term “I don’t know” was salient across all three medical groups in that participants either stated “I don’t know” as a free listing response or began their response with “I don’t know/I’m not sure if e-cigarettes cause ________.” For residents and medical students, the idea of e-cigarettes being “less harmful than cigarettes” was salient, while nursing students associated e-cigarettes with having the “same risks as smoking”. “Lung cancer” was salient among both medical students and nursing students. Nursing students also associated e-cigarettes with heart disease. Terms uniquely salient to attending physicians included “nicotine” and “chemicals.”

### Comparing knowledge of E-cigarettes and health impacts of use

**Fig. 4:**
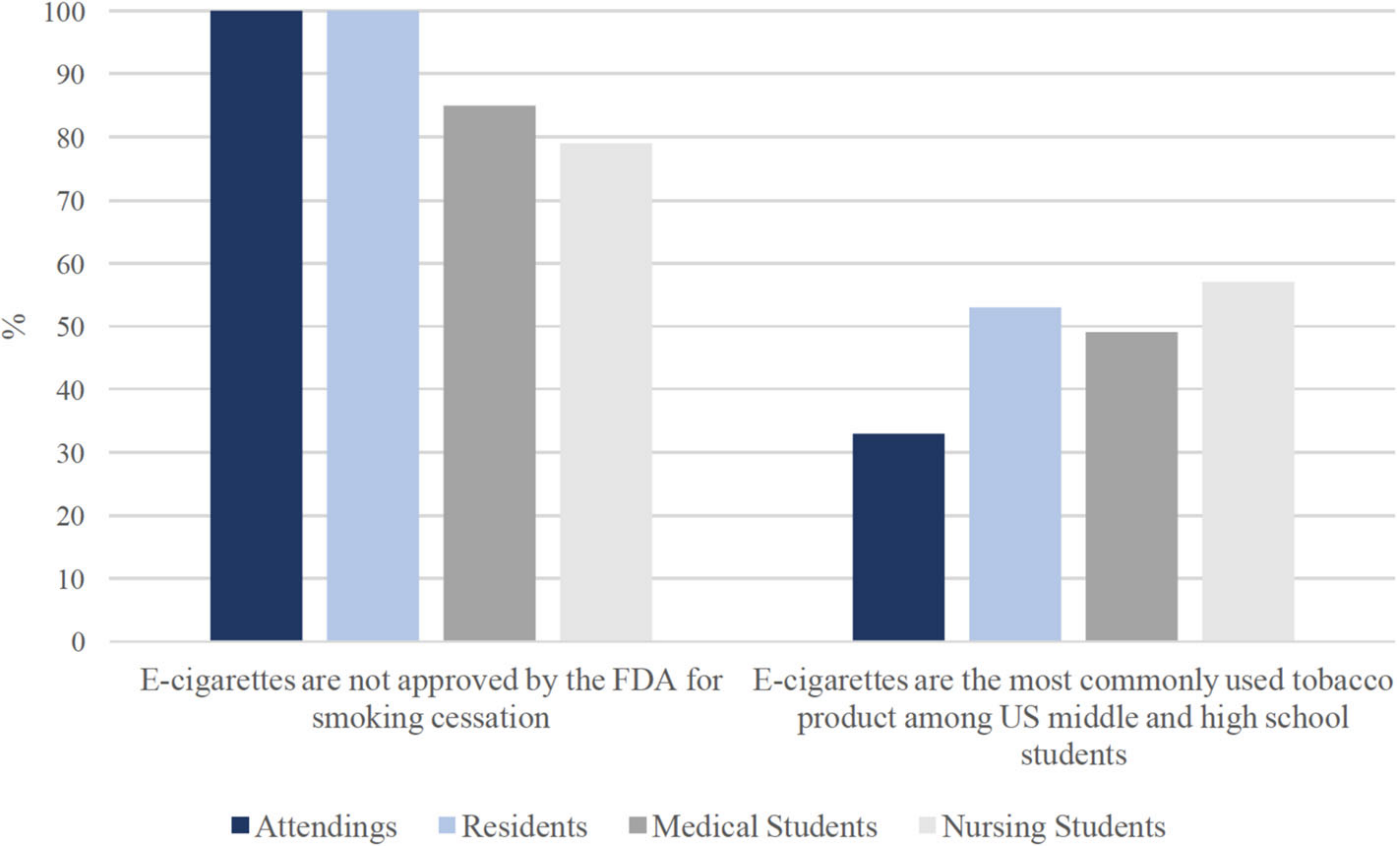
Differences by occupation in the rate of correct responses to forced-choice survey items

Participants’ responses to the knowledge-based survey components are summarized in Table 2. Percentage responding correctly to each forced-choice question among occupation group is shown in Fig. 4. All of the attending and resident physicians interviewed answered correctly that e-cigarettes are not FDA-approved as a tobacco cessation product. Among medical and nursing students, 85 and 79%, respectively, answered correctly. When asked to choose which tobacco product is used most commonly by U.S. middle and high school students, nursing students had the highest percentage of respondents answering correctly (57%) that e-cigarettes are the most common, compared 33% of attendings.

**Table 2:** Comparing survey responses among occupational groups

		Attendings (N = 15)	Residents (*N* = 15)	Medical Students (N = 33)	Nursing Students (N = 28)
Forced-Choice Items		n (%)			
E-cigarettes are not approved by the FDA for smoking cessation.	*Correct*	15 (100)	15 (100)	28 (85)	22 (79)
E-cigarettes are the most commonly used tobacco product among US middle and high school students.	*Correct*	5 (33)	8 (53)	16 (49)	16 (57)
Likert-Type Items		n (%)			
Among youth and young adults, the use of e-cigarettes is associated with subsequent initiation of cigarette use.	*Correct*	11 (73)	8 (53)	14 (42)	22 (79)
	*Did not know*	4 (27)	2 (13)	15 (46)	2 (7)
Nicotine intake from e-cigarettes can be comparable to that from cigarettes.	*Correct*	8 (53)	9 (60)	18 (55)	17 (61)
	*Did not know*	4 (27)	4 (27)	5 (15)	5 (18)
Exposure to toxicants and carcinogens from e-cigarettes is significantly lower compared to conventional cigarettes.	*Correct*	9 (60)	6 (40)	16 (49)	6 (22)
	*Did not know*	4 (27)	5 (33)	6 (18)	7 (26)

**Fig. 5:**
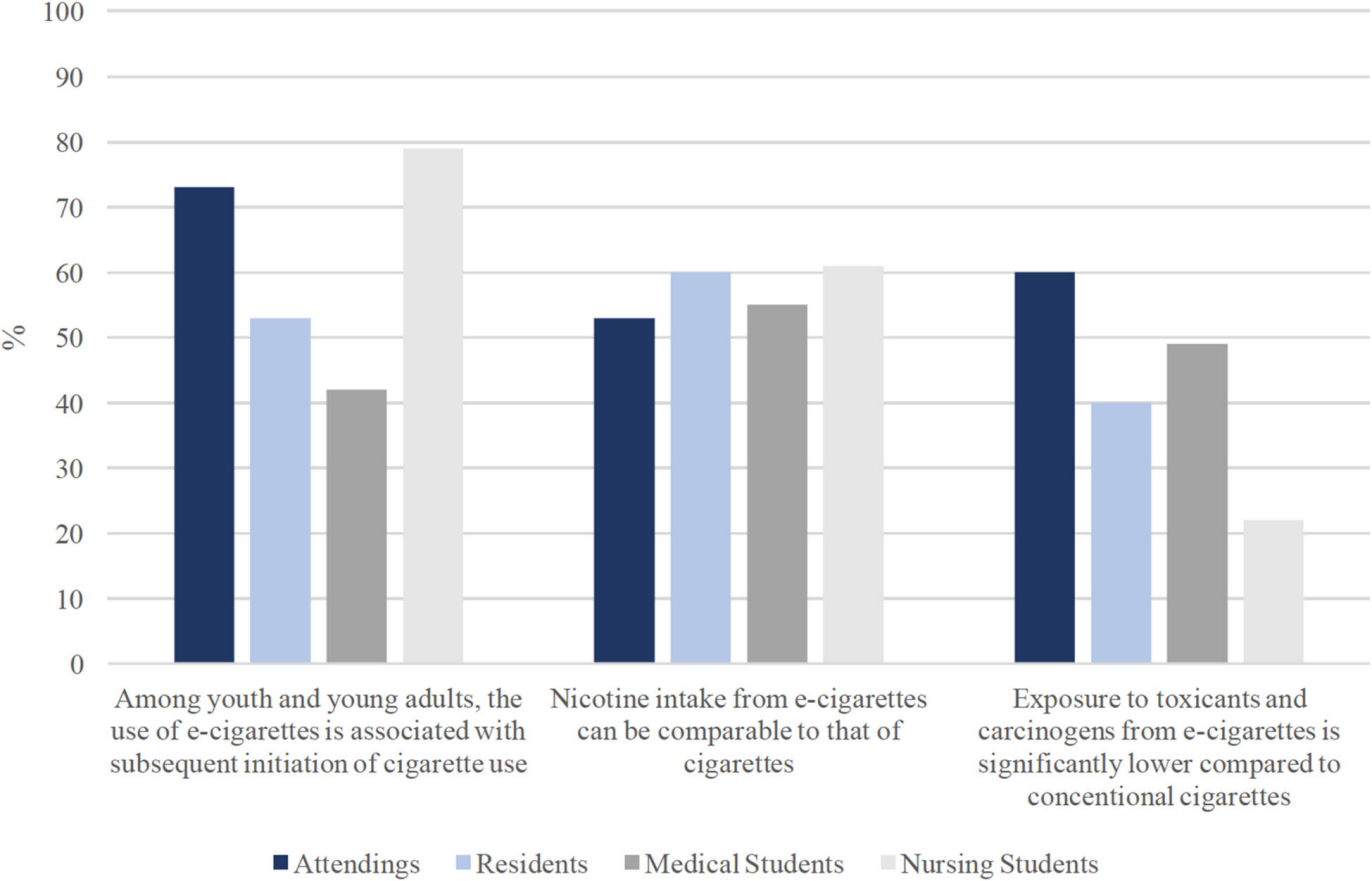
Differences by occupation in the rate of correct responses to Likert-type survey items

**Fig. 6:**
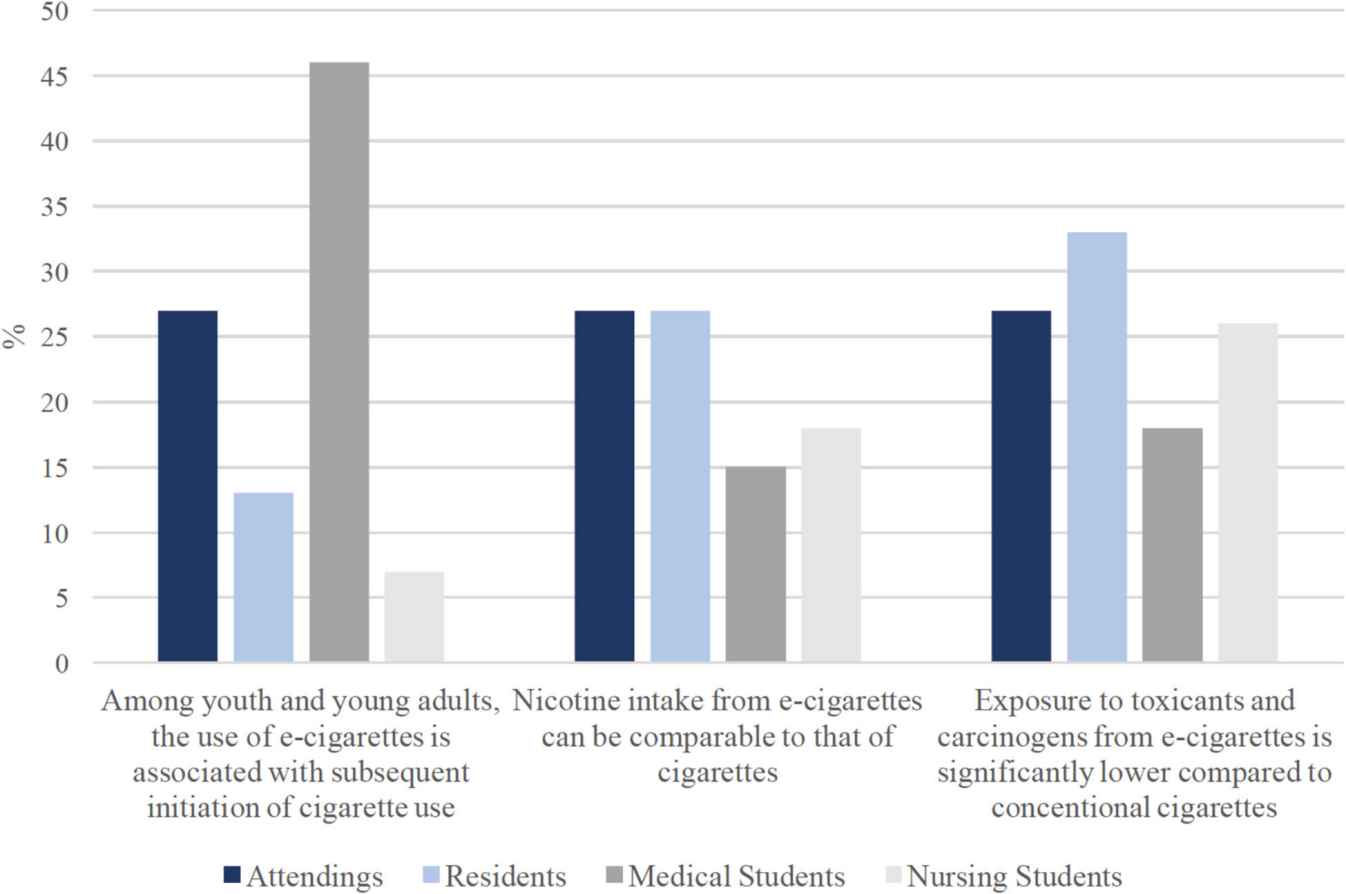
Differences by occupation in the rate of participants responding “I don’t know” to Likert-type survey items

Percentage of correct responses to Likert-type questions among each occupation is shown in Fig. 5. Percentage of respondents choosing “I don’t know” to Likert-type questions among each occupation is shown in Fig. 6. When asked about the association between e-cigarette use and subsequent initiation of cigarette use, 79% of nursing students who were interviewed responded correctly, compared to 42% of medical students, with a similar proportion of medical students (46%) responded that they did not know. When asked about nicotine intake from e-cigarettes compared with cigarettes, again nursing students had the highest percentage of respondents answering correctly (61%), compared to 55% of medical students, and 53% of attendings. When asked about general exposure of e-cigarette-only users to toxicants and carcinogens compared to cigarette-only users, one in three residents interviewed responded that they did not know, as did 27% of attendings, 26% of nursing students, and 18% of medical students.

The sources of information regarding e-cigarettes is summarized in Table 3. Among attending physicians, news outlets were the most common source of e-cigarette-related information. For resident physicians and nursing students, social media was the most common source of e-cigarette-related information. Medical students reported that news outlets and social media were the most common sources of e-cigarette-related information.

**Table 3:** Percent of participants who have learned about e-cigarettes through various sources^a^

Source	Attendings (N = 15)	Residents (N = 15)	Medical Students (N = 33)	Nursing Students (N = 28)
	n (%)			
Occupation ^b^	8 (53)	2 (13)	10 (30)	6 (21)
Medical journals	7 (47)	4 (27)	3 (9)	0 (0)
Evidence-based summaries ^c^	3 (20)	1 (7)	1 (3)	4 (14)
News outlets	11 (73)	8 (53)	19 (58)	11 (39)
Social media	7 (47)	10 (67)	19 (58)	19 (68)
Magazines and ads	7 (47)	4 (27)	10 (30)	11 (39)
Conversation with e-cigarette non-users	3 (20)	5 (33)	13 (39)	5 (18)
Conversation with e-cigarette users	4 (27)	8 (53)	13 (39)	17 (61)

^a^Responses are not mutually exclusive

^b^ Occupational sources include lectures, conferences, and meetings

^c^Evidence-based summaries include Surgeon General’s reports, and consensus study reports

## Discussion

Current and future healthcare professionals lack an evidence-based framework with which to approach health implications of e-cigarette use, to the potential detriment of patient counseling and education. This study allowed us to better understand the knowledge baseline and differences in lexicon among different participant groups, highlighting important opportunities for educational interventions tailored to respective learners.

Between-group variation in salient freelisting response point to differences in personal experience at varied levels of training. Terms traditionally associated with combustible cigarettes were more commonly used by attending physicians, suggesting that their understanding of e-cigarettes is nested primarily in their knowledge of tobacco, perhaps because they have had longer exposure to traditional cigarette smoking prevention efforts compared to more junior trainees. We found that residents, medical students, and nursing students were more likely to comment on product design, using language unique to e-cigarettes. This suggests that younger healthcare professionals may have greater familiarity with these devices compared to attending physicians. Notably, the terms that were salient across all groups, “young people” and “smell,” are anchored in day-to-day observations rather than a professional health care perspective. In fact, participants in the medical field often began their response with the words “I don’t know” when asked about the health implications of e-cigarette use, stating that the health effects are “not well understood”. Overall, our findings show that participants in the undergraduate and post-graduate levels of training lack a standardized health-oriented approach when speaking about e-cigarettes. This is concerning as these discrepancies may translate to variation information and recommendations that healthcare professionals provide to patients.

Results from forced-choice survey items reveal that misinformation about e-cigarettes exists at a fundamental level, which can misinform patient counseling. All respondents reported a lack of familiarity with trends in tobacco use among youth. The majority of attending physicians were under the false understanding that cigarettes were the most common tobacco product used by youth. In reality, e-cigarette use among middle- and high-school students surpassed cigarette use in 2014 and has remained in the lead since [43]. These findings suggest that younger healthcare providers (residents and students) may be more aware of the prevalence of e-cigarettes. Among our survey respondents, practicing physicians (residents and attendings) were all aware that e-cigarettes are not approved by the FDA for smoking cessation. However, 15% of medical students and 21% of nursing students falsely believed that these devices do have FDA approval for smoking cessation, consistent with prior work [43]. This highlights the urgent need for education of basic public health policies pertinent to clinical practice at the undergraduate level of training.

Familiarity with evidence-based health implications and risks associated with e-cigarette use did not correlate with level of training. For example, research has shown nicotine levels in e-cigarettes to be highly variable due to lack of manufacturing and labelling regulations, to the point that nicotine intake from e-cigarettes can be comparable to that of cigarette users [5, 7, 22]. In our study, 30% of medical students disagreed with this statement and 15% chose “I don’t know.” Even at the attending level, over a quarter of attendings interviewed chose “I don’t know” rather than choosing to agree or disagree with these evidence-based survey items. This suggests that both undergraduate and postgraduate professionals lack baseline understanding of some of the most researched health implications of e-cigarette use. Prior literature with similar findings note that lack of knowledge is a barrier to speaking with patients and providing them with informed medical counseling [30, 33–35].

Our study indicates that a contributing factor to the variability in lexicon and baseline knowledge is that participants seem to anchor their understanding on personal experiences with the product rather than evidence based sources such as classroom didactics, continuing medical education (CME) training, or medical journals. 53% of attendings received e-cigarette-related information through professional training (e.g. meetings, lectures), compared to 13% of residents, 30% of medical students, and 18% of nursing students. It is common practice for physicians to supplement their knowledge and stay up-to-date on research through literature. Yet an even lower proportion of participants from each group reported reading about e-cigarettes in evidence-based summaries, such as the 2016 Surgeon General’s Report or the 2018 consensus study report by the National Academies of Sciences, Engineering, and Medicine [3, 22]. These reports are publicly accessible and summarize the current evidence on the health consequences of e-cigarette use in an organized way. Instead, the most common source of information for participants in our study were news outlets and social media, tailored for the general public rather than medical professionals. These findings are consistent with previous studies demonstrating that healthcare professionals learn about e-cigarettes primarily from anecdotal sources (patients, news, advertisements) as opposed to professional training [35, 42, 43]. Despite the growing body of research on the health impacts associated with e-cigarette use, this knowledge is not reaching healthcare professionals who are uniquely positioned at the interface of the community, scientific research, and public health.

A limitation of our study is that findings represent the responses from the convenience sampling of students, residents, and attendings from a single medical center. Additionally, smaller number of attending and resident physicians than anticipated participated in the study. Although free listing studies have no threshold for adequate sample size, prior literature suggests a minimum of 20 participants per group [36]. With the low number of participants, we did not further stratify medical and nursing student groups by year of training as we would not be able to reach saturation when analyzing the data. Because there is a wide range in years of clinical experience among graduate medical professionals that may moderate the responses, future studies should aim to achieve a larger sample size. Finally, standardization of free listing terms during data cleaning may have been subject to bias in interpreting meaning and categorizing synonymous ideas. Effort was made to reduce bias by having two researchers involved in the standardizing the responses.

Our study showed that there is ultimately a lack of common, scientifically-driven understanding of e-cigarettes among healthcare professionals, even though reliable sources of information are available. If even providers are not up-to-date with the current research on e-cigarettes, having an informed public will be an even greater challenge. To ensure that providers are providing patients with evidence-based knowledge, curricular development is needed at not only the undergraduate level but the graduate level as well. Both medical schools and residency programs should integrate e-cigarette education into an existing curriculum on nicotine addiction. We recommend that this curriculum highlight the current literature and establish an evidence-based framework for providers effectively communicate and counsel patients on the health impacts of e-cigarette use.

## Conclusion

In conclusion, this study provides valuable insight into the variable perception of e-cigarettes among healthcare professionals at different levels of training that may impact the quality of patient education on this subject. Furthermore, it identifies specific gaps in knowledge among students and practicing physicians regarding health consequences of e-cigarette use. It is critical that medical professionals stay abreast of the scientific studies on alternative tobacco products, especially as these trends continue to change over time. It is time that formal education about e-cigarettes and their impact on human health be incorporated into each phase of medical training, from undergraduate medical curriculum to residency training to continuing medical education.

## Supplementary information

**Additional file 1.** Data collection survey questionnaire used to assess participants’ knowledge of electronic cigarettes and associated health consequences of use.

**Additional file 2 **Comparing participants’ perspectives on e-cigarettes by occupational group*.* Table of results generated from free listing data analysis.

## Data Availability

Not applicable.

## Abbreviations

e-cigarettes: Electronic cigarettes
CME: Continuing medical education
TJU: Thomas Jefferson University
TJUH: Thomas Jefferson University Hospital
ENDS: Electronic Nicotine Delivery Systems
FDA: U.S. Food and Drug Administration
c-cigarettes: Combustible cigarettes
IRB: Institutional Review Board

## References

[1] Giovenco DP, Hammond D, Corey CG, Ambrose BK, Delnevo CD (2015). E-cigarette market trends in traditional U.S. retail channels, 2012-2013. Nicotine Tob Res.

[2] Zhu S-H, Sun JY, Bonnevie E, Cummins SE, Gamst A, Yin L (2014). Four hundred and sixty brands of e-cigarettes and counting: implications for product regulation. Tob Control.

[3] U.S. Department of Health and Human Services (2016). E-Cigarette Use Among Youth and Young Adults: A Report of the Surgeon General-Executive Summary.

[4] Jenco M (2018). Study: Youth tobacco use decreasing; e-cigarettes most popular. AAP News.

[5] Peace MR, Baird TR, Smith N, Wolf CE, Poklis JL, Poklis A (2016). Concentration of nicotine and glycols in 27 electronic cigarette formulations. J Anal Toxicol.

[6] The Truth Initiative (2018). E-cigarettes: Facts, stats and regulations.

[7] Dawkins LE, Kimber CF, Doig M, Feyerabend C, Corcoran O (2016). Self-titration by experienced e-cigarette users: blood nicotine delivery and subjective effects. Psychopharmacology.

[8] U.S. Department of Health and Human Services (2014). The Health Consequences of Smoking—50 Years of Progress: A Report of the Surgeon General.

[9] Treur JL, Willemsen G, Bartels M, Geels LM, van Beek JHDA, Huppertz C (2015). Smoking during adolescence as a risk factor for attention problems. Biol Psychiatry.

[10] Glantz SA (2018). Even more evidence for a gateway effect for e-cigs to cigarette smoking, this time from Germany.

[11] Young-Wolff KC, Klebaner D, Folck B, Tan ASL, Fogelberg R, Sarovar V (2018). Documentation of e-cigarette use and associations with smoking from 2012 to 2015 in an integrated healthcare delivery system. Prev Med.

[12] Miech R, Patrick ME, O’Malley PM, Johnston LD (2017). E-cigarette use as a predictor of cigarette smoking: results from a 1-year follow-up of a national sample of 12th grade students. Tob Control.

[13] Soneji S, Barrington-Trimis JL, Wills TA, Leventhal AM, Unger JB, Gibson LA (2017). Association between initial use of e-cigarettes and subsequent cigarette smoking among adolescents and Young adults: a systematic review and meta-analysis. JAMA Pediatr.

[14] Williams M, Villarreal A, Bozhilov K, Lin S, Talbot P (2013). Metal and silicate particles including nanoparticles are present in electronic cigarette cartomizer fluid and aerosol. PLoS One.

[15] Ganapathy V, Manyanga J, Brame L, McGuire D, Sadhasivam B, Floyd E (2017). Electronic cigarette aerosols suppress cellular antioxidant defenses and induce significant oxidative DNA damage. PLoS One.

[16] Olmedo P, Goessler W, Tanda S, Grau-Perez M, Jarmul S, Aherrera A (2018). Metal concentrations in e-cigarette liquid and aerosol samples: the contribution of metallic coils. Environ Health Perspect.

[17] Gerloff J, Sundar IK, Freter R, Sekera ER, Friedman AE, Robinson R (2017). Inflammatory response and barrier dysfunction by different e-cigarette flavoring chemicals identified by gas chromatography-mass spectrometry in e-liquids and e-vapors on human lung epithelial cells and fibroblasts. Appl In Vitro Toxicol.

[18] Chun LF, Moazed F, Calfee CS, Matthay MA, Gotts JE (2017). Pulmonary toxicity of e-cigarettes. Am J Physiol Lung Cell Mol Physiol.

[19] Ghosh A, Coakley RC, Mascenik T, Rowell TR, Davis ES, Rogers K (2018). Chronic E-cigarette exposure alters the human bronchial epithelial proteome. Am J Respir Crit Care Med.

[20] Bayly JE, Bernat D, Porter L, Choi K (2019). Secondhand exposure to aerosols from electronic nicotine delivery systems and asthma exacerbations among youth with asthma. Chest..

[21] Farsalinos KE, Polosa R (2014). Safety evaluation and risk assessment of electronic cigarettes as tobacco cigarette substitutes: a systematic review. Ther Adv Drug Saf.

[22] National Academies of Sciences, Engineering, and Medicine (2018). Public Health Consequences of E-Cigarettes.

[23] Bullen C, Howe C, Laugesen M, McRobbie H, Parag V, Williman J (2013). Electronic cigarettes for smoking cessation: a randomised controlled trial. Lancet.

[24] Hartmann-Boyce J, McRobbie H, Bullen C, Begh R, Stead LF, Hajek P (2016). Electronic cigarettes for smoking cessation. Cochrane Database Syst Rev.

[25] Manzoli L, Flacco ME, Ferrante M, La Vecchia C, Siliquini R, Ricciardi W (2017). Cohort study of electronic cigarette use: effectiveness and safety at 24 months. Tob Control.

[26] Rigotti NA, Chang Y, Tindle HA, Kalkhoran SM, Levy DE, Regan S (2018). Association of E-cigarette use with smoking cessation among smokers who plan to quit after a hospitalization: a prospective study. Ann Intern Med.

[27] Kulik MC, Lisha NE, Glantz SA (2018). E-cigarettes associated with depressed smoking cessation: a cross-sectional study of 28 European Union countries. Am J Prev Med.

[28] Hajek P, Phillips-Waller A, Przulj D, Pesola F, Myers Smith K, Bisal N (2019). A randomized trial of E-cigarettes versus nicotine-replacement therapy. N Engl J Med.

[29] Shin DW, Kim YI, Kim SJ, Kim JS, Chong S, Park YS (2017). Lung cancer specialist physicians’ attitudes towards e-cigarettes: a nationwide survey. PLoS One.

[30] Bascombe TMS, Scott KN, Ballard D, Smith SA, Thompson W, Berg CJ (2016). Primary healthcare provider knowledge, beliefs and clinic-based practices regarding alternative tobacco products and marijuana: a qualitative study. Health Educ Res.

[31] Hiratsuka VY, Avey JP, Trinidad SB, Beans JA, Robinson RF (2015). Views on electronic cigarette use in tobacco screening and cessation in an Alaska native healthcare setting. Int J Circumpolar Health.

[32] Kandra KL, Ranney LM, Lee JGL, Goldstein AO (2014). Physicians’ attitudes and use of e-cigarettes as cessation devices, North Carolina, 2013. PLoS One.

[33] Nickels AS, Warner DO, Jenkins SM, Tilburt J, Hays JT (2017). Beliefs, practices, and self-efficacy of US physicians regarding smoking cessation and electronic cigarettes: a National Survey. Nicotine Tob Res.

[34] Gorzkowski JA, Whitmore RM, Kaseeska KR, Brishke JK, Klein JD (2016). Pediatrician knowledge, attitudes, and practice related to electronic cigarettes. J Adolesc Health.

[35] Pepper JK, McRee A-L, Gilkey MB (2014). Healthcare providers’ beliefs and attitudes about electronic cigarettes and preventive counseling for adolescent patients. J Adolesc Health.

[36] Weller SC, Romney AK. Qualitative Research Methods: Systematic data collection. Newbury Park: SAGE Publications; 1988. 10.4135/9781412986069.

[37] Fiks AG, Gafen A, Hughes CC, Hunter KF, Barg FK (2011). Using freelisting to understand shared decision making in ADHD: parents’ and pediatricians’ perspectives. Patient Educ Couns.

[38] Dress EM, Frasso R, Calkins ME, Curry AE, Kohler CG, Schmidt LR (2018). Comparing patient, clinician, and caregiver perceptions of care for early psychosis: a free listing study. Narrat Inq Bioeth.

[39] Sobo EJ, Kurtin P (2003). Variation in physicians’ definitions of the competent parent and other barriers to guideline adherence: the case of pediatric minor head injury management. Soc Sci Med.

[40] Frasso R, Keddem S, Golinkoff JM, Cnaan RA, Milofsky C (2018). Qualitative methods: tools for understanding and engaging communities. Handbook of community movements and local organizations in the 21st century.

[41] Schrauf RW, Sanchez J (2008). Using freelisting to identify, assess, and characterize age differences in shared cultural domains. J Gerontol B Psychol Sci Soc Sci.

[42] Barg FK, Keddem S, Ginsburg KR, Winston FK (2009). Teen perceptions of good drivers and safe drivers: implications for reaching adolescents. Inj Prev.

[43] Jamal A, Gentzke A, Hu SS, Cullen KA, Apelberg BJ, Homa DM (2017). Tobacco use among middle and high school students - United States, 2011-2016. MMWR Morb Mortal Wkly Rep.

