# Supplementary material for: Where there’s smoke, there’s fire: what current and future providers do and do not know about electronic cigarettes
Source: BMC Public Health. 2020 Jul 20;20:1145. doi: 10.1186/s12889-020-09265-5 (PMC7372802; doi:10.1186/s12889-020-09265-5)
Supplement: Supplementary file 1 — Additional file 1. Data collection survey questionnaire used to assess participants’ knowledge of electronic cigarettes and associated health consequences of use. [file 12889_2020_9265_MOESM1_ESM.pdf]

DATA COLLECTION FORM # ____________

FREELISTING – *TO BE FILLED OUT BY THE INTERVIEWER*

Interviewer: I'll be asking you to list your thoughts for me. For example, I'll ask a question like "List all the words that come to mind when you think about driving. You might say "Traffic, cars, pedestrians"…etc. I will write down what your responses until you can't think of any more words.
Do you have any questions for me? Okay, now—

1. List all the words that come to mind when you of e-cigarettes.	2. List all the health implications that come to mind when you about e-cigarette use.

Interviewer Transition: Thank you. Now I would like you to fill out this brief survey.
Turn over this page and hand it to the participant along with a pen. Please stay with the participant but respect their privacy as he/she fills out the survey.

DATA COLLECTION FORM # ____________

SURVEY – *TO BE FILLED OUT BY THE PARTICIPANT*

1. Please select your age group.
   - ☐ 18-25
   - ☐ 26-30
   - ☐ 31-35
   - ☐ 36-40
   - ☐ 41-45
   - ☐ 46-50
   - ☐ 51-55
   - ☐ 56-60
   - ☐ 61-65
   - ☐ 66-70
   - ☐ 71 and older
2. Which of the following best describes your position?
   - ☐ Attending physician
   - ☐ Resident physician
   - ☐ Intern
   - ☐ Fellow (post-residency)
   - ☐ Nurse/Nurse practitioner
   - ☐ Physician's assistant (PA)
   - ☐ Medical student
   - ☐ Nursing student
   - ☐ PA student
   - ☐ Other: ________________________
3. From your perspective, which tobacco product is most commonly used by US middle and high school students?
   - ☐ Cigarettes
   - ☐ Cigars/cigarillos
   - ☐ Smokeless tobacco (eg. snuff, dip, chewing tobacco)
   - ☐ Hookah
   - ☐ Electronic cigarettes
4. E-cigarettes are FDA-approved smoking cessation products.
   - ☐ True
   - ☐ False

**To what degree do you agree with the following statements?** *(Select one option per row)*	**I don't know**	**Strongly disagree**	**Disagree**	**Agree**	**Strongly agree**
5. Among youth and young adults (ages 15-24), the use of e-cigarettes is strongly associated with subsequent initiation of cigarette use.	☐	☐	☐	☐	☐
6. E-cigarette-only users are exposed to lower levels of nicotine compared to cigarette-only users.	☐	☐	☐	☐	☐
7. E-cigarette-only users are exposed to lower levels of toxicants and carcinogens compared to cigarette-only users.	☐	☐	☐	☐	☐

8. Where have you learned about e-cigarettes? *(Select all that apply)*
   - ☐ E-cigarette non-users
   - ☐ E-cigarette users
   - ☐ News outlets
   - ☐ Occupation (eg. lectures, conferences, meetings)
   - ☐ Medical journals
   - ☐ Evidence-based summaries and/or reports (eg. Surgeon General's reports)
   - ☐ Magazines /Advertisements
   - ☐ Social media
   - ☐ Other ______________________________________
